# Formulation, Characterization and Cytotoxic Effect of Indomethacin-loaded Nanoparticles

**DOI:** 10.2174/0118715230348349241126053733

**Published:** 2024-12-27

**Authors:** Kaan Yalçınkaya, Behiye Şenel, Evrim Akyıl

**Affiliations:** 1 Department of Pharmaceutical Technology, Faculty of Pharmacy, Anadolu University, Eskişehir, Turkey;; 2 Department of Pharmaceutical Biotechnology, Faculty of Pharmacy, Anadolu University, Eskişehir, Turkey

**Keywords:** Indomethacin, polymeric nanoparticles, solid lipid nanoparticles, nanoprecipitation, homogenization, dynasan^®^116

## Abstract

**Background:**

Indomethacin (IND), classified as class 2 in the Biopharmaceutical Classification System (BCS), has emerged as an anti-inflammatory agent with low solubility and high permeability. Widely used in the treatment of various diseases, such as rheumatoid arthritis and ankylosing spondylitis, this drug is well-known for its adverse effects, particularly in the stomach, and a short biological half-life, which is around 1.5-2 hours.

**Objectives:**

The aim of this study was to overcome the challenges of low solubility, short half-life, and serious side effects occurring with the use of IND-loaded formulations of Solid Lipid Nanoparticles (SLNs) and Polymeric Nanoparticles (PNPs).

**Methods:**

For PNPs, emulsification/solvent evoporation method was employed, and for SLNs, the hot homogenizaton method was applied. Eudragit^®^ RLPO (RLPO) and Eudragit® RSPO (RSPO) were used as polymers for PNP and Dynasan^®^116 (DYN) was used as the solid lipid for SLN. Prepared formulations were characterized for Particle Size (PS), Polydispersity Index (PDI), Zeta Potential (ZP), Encapsulation Efficiency (%EE), and drug-excipient compatibility using DSC, FT-IR, and ^1^H NMR; cumulative drug release rates were assessed using HPLC and *in vitro* cytotoxicities were examined by the MTT assay.

**Results:**

Both PNP and SLN formulations’ zeta potential, particle size, and PDI results indicated the formulations to have good stability. Encapsulation efficiency values were obtained as desired. Drug-excipient compatibility was proved using DSC, FT-IR, and ^1^H NMR. *In vitro* dissolution results have proven both formulations to have longer release than pure indomethacin. In the MTT analysis of indomethacin application for 24 and 48 hours, a linear correlation was observed between drug concentration and cell viability, and it was determined that the PNP formulation exhibited fewer toxic effects among the formulations. This has proven the PNP nanocarrier as safer for normal cells.

**Conclusion:**

IND-loaded PNP and SLN formulations have been successfully prepared in this work and they have achieved drug release in the intestine and prolonged the release duration.

## 1 INTRODUCTION

Indomethacin (IND) is an analgesic, antipyretic, and anti-inflammatory drug (NSAID), which is widely used for the treatment of severe diseases, including acute gout, ankylosing spondylitis, rheumatoid arthritis, and osteoarthritis [1]. However, according to the formal reports, serious side effects, such as gastrointestinal hemorrhage, peptic ulcer, and digestive problems can be seen after long-term IND usage [2]. In addition, IND has an adequately short half-life, which leads to dosing at least 2 or 3 times a day [3].

The use of nanoparticle drug delivery systems has increased further due to problems arising from the low solubility of almost half of the new active substances introduced to the market. Drugs loaded into nanoparticles are protected from environmental factors and have enhanced solubility with the matrix containing nanoparticle components. In addition, they reduce the risk of possible side effects of traditional dosage forms and reduce the toxic effects of drugs by being suitable for targeting and being able to eliminate the first-pass effect [4].

Polymeric Nanoparticles (PNPs) are defined as nano-sized drug delivery systems containing particles with a size of 10-100 nanometers [5]. PNPs consist of two types: nanospheres and nanocapsules. The advantages of PNPs can be considered as their potential to provide controlled release, protect the drug from the biological environment, and improve the bioavailability of the drug [6].

Solid Lipid Nanoparticles (SLNs) are colloidal systems developed for lipophilic drugs with low solubility, containing particles with a size of 50-1000 nanometers [7]. The major advantage of SLNs is that they involve the advantages of the traditional drug delivery systems (such as emulsions, suspensions, and liposomes) and do not include their disadvantages. In addition, improving drug solubility, modifying the drug release, and providing large-scale production can be considered as the advantages of SLNs [8].

The aim of this study was to develop and characterize PNPs and SLNs to minimize their side effect profile and increase the dosing interval of indomethacin. Therefore, polymers and lipids resistant to gastric pH, such as Eudragit^®^ RLPO, Eudragit^®^ RSPO, and Dynasan^®^116, have been used to develop the nanoparticles.

## 2 MATERIALS AND METHODS

IND was kindly gifted by Deva, Turkey. Eudragit^®^ RLPO and Eudragit^®^ RSPO were purchased from Röhm, Germany. Tween^®^80 and Span^®^80 were obtained from Merck, Germany. The water used in all experiments was freshly collected from the Agilent water purification system (Agilent, Germany). All other chemicals used were of analytical grade.

### 2.1 Preparation of Nanoparticles

#### 2.1.1 Preparation of PNPs

Emulsification/solvent evaporation method was used to prepare the PNP formulation [9]. Different ratios of RLPO: RSPO polymers were used in order to choose the formulation with the best particle size, PDI, and zeta potential properties. The composition of three different formulations is given in Table **1**.

#### 2.1.2 Preparation of SLNs

Hot homogenization method was used to prepare the SLN formulation [10]. Different ratios of surfactants were used in order to get smaller particle size and PDI and the highest value of zeta potential. The composition of SLN formulations is given in Table **2**.

### 2.2 Physiochemical characterization of Nanoparticles

#### 2.2.1 Particle Size (PS), PDI, and Zeta Potential (ZP)

Malvern Zetasizer Nano-ZS (England) was used to obtain the measurements of PS, PDI, and ZP. Samples were prepared by dilution of PNP and SLN dispersions in bidistilled water. All formulations were analyzed at 25°C.

#### 2.2.2 Differential Scanning Calorimetry (DSC)

The physical states of the formulations were characterized by using the Shimadzu DSC-60 instrument (Japan). Aluminum cells with ~4 mg samples were analyzed under the atmosphere of nitrogen gas (50 mL/min) and a heating rate of 10°C/min within a temperature range of 30-300°C. Pure IND and excipients were also analyzed.

#### 2.2.3 Fourier Transform Infrared (FT-IR) Spectrophotometry

FT-IR spectra of the formulations were obtained by using the Shimadzu IR Prestige-21 (Japan) instrument at the range of wavelength of 4000-500 cm^-1^. Pure IND and excipients were also analyzed.

#### 2.2.4 Nuclear Magnetic Resonance (NMR)

^1^H NMR analyses were characterized by using UltraShield™ CPMAS NMR instrument (Brucker, Germany). Samples were prepared by dissolving formulations and pure ingredients in deuterated chloroform.

#### 2.2.5 Encapsulation Efficiency (%EE)

HPLC (Shimadzu-20 A, Japan) was used for the determination of IND. Analyses were conducted using a C8 column (150 × 4.6 mm, 5µm) at 25°C. The mobile phase included a mixture of 2.5% (v/v) o-phosphoric acid:methanol: acetonitrile (2:1:2; v/v). Analyses were carried out at a flow rate of 1 mL.min^-1^ with an injection volume of 20 µL and detection wavelength at 240 nm [11].

### 2.3 *In Vitro* Drug Release Test

Two tests were performed; for the first test, in the first two hours, membranes were immersed in the media containing 0.1N HCl (pH 1,2) and PBS (pH 6,8), and for the second test, PBS (pH 6,8) and 1% Tween^®^ 80 were used as the media. The test media were 50 mL each at 37°C ± 1°C, with the speed set at 100 rpm. Samples were taken at the predetermined time intervals and analyzed using HPLC. Then, the cumulative drug release percentage was calculated.

### 2.4 MTT Assay

The effects of pure IND, PNP, and SLN on cell viability were assessed using the MTT assay. For this purpose, IND solutions were first prepared at concentrations ranging from 1 μM to 100 μM, and the corresponding amounts of these concentrations in the formulation were calculated. In the analysis, HUVEC cells were treated with pure IND, blank formulations, and IND-loaded formulations to determine cell viability values at 24 and 48 hours. The effects of the studied groups on cell viability were evaluated based on time and concentration, and the results were presented graphically with statistical analyses. The analyses were conducted in triplicate, and the results were evaluated using GraphPad Prism 9.0 software. The IC_50_ values for the effect of each group on the cells were also calculated.

## 3 EXPERIMENTAL

### 3.1 Preparation of PNP

The required amount of the polymer or mixture of polymers and IND was weighed and dissolved in 5mL Dichloromethane (DCM) under continuous stirring using a high-speed homogenizer (Heidolph, Germany); the mixture was then added into water and Tween^®^80 solution in terms of droplets. Then, after obtaining an emulsion, DCM was evaporated using a magnetic stirrer (IKA, Germany) inside a fume cupboard (Hedlab, Turkey) and the formulation was freeze-dried to get nanoparticles in the powder form.

### 3.2 Preparation of SLN

For investigating the optimum lipid, the same formulation was prepared with 5 different lipids (Dynasan^®^114, Dynasan^®^116, Compritol^®^ 888 ATO, Gelucire^®^ 44/14, Precirol^®^ ATO 5) without using any surfactants. Briefly, lipid and IND were heated up to 5°C above the lipid’s melting point and the mixture was added to the aqueous phase with the same temperature, and homogenization was carried out using a high-speed homogenizer. After cooling to room temperature, the supernatant was taken and analyzed. The lipid of the sample with the lowest amount of IND was chosen as the optimum lipid. The amount of IND was analyzed according to the formula given in Eq. (1).


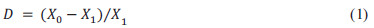


Where, D is the value of IND loaded in the lipid, X_0_ is the amount of IND fed to the system, and X_1_ is the amount of IND analyzed in the supernatant [8].

For the formulation, the same process was carried out using Span^®^80 in the oily phase and Tween^®^80 in the aqueous phase. After homogenization, the formulations were freeze-dried to get nanoparticles in powder form.

### 3.3 Encapsulation Efficiency (%EE) and Loading Efficiency (%LE)

For the calculation of PNP’s %EE, 5 mg of the formulation was weighted and dissolved in 1 mL of mobile phase in an ultrasonic bath (Wisd Laboratory Instruments, South Korea); it was then analyzed using the formula given in Eq. (2).


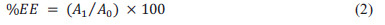


Where, A_1_ is the amount of IND analyzed and A_0_ is the total amount of IND fed to the system [12].

For the calculation of SLN’s %LE, 10 mg of the formulation was weighed and added to 1 mL of methanol in an ultrasonic bath and the unencapsulated IND was dissolved. Then the mixture was centrifuged and the supernatant was analyzed using the formula given in Eq. (3).


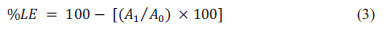


Where, A_1_ is the amount of IND analyzed and A_0_ is the total amount of IND fed to the system [13].

## 4 RESULTS AND DISCUSSION

### 4.1 Optimal PNP Formulation

Eudragit^®^ RLPO and Eudragit^®^ RSPO were used as polymers. Both are pH-independent non-biodegradable polymers and are known to prolong the drug release time [14, 15]. As the optimum formulation, the P-2 coded formulation was chosen, as given in Table **1**, in which both polymers were used in a ratio of 1:1 [16].

### 4.2 Optimal SLN Formulation

D values of the lipids used are given in Fig. (**1**).

According to the D values calculated, Dynasan^®^116 was chosen as the optimum lipid.

### 4.3 Physiochemical Characterization of Nanoparticles

#### 4.3.1 Particle Size (PS), PDI, and Zeta Potential (ZP)

A reduction in particle size enhances the surface area, thus improving the solubility and bioavailability of poorly water-soluble drugs [17]. ZP values indicate the charge on the surface of the nanoparticles [18]. Surface charge is a remarkable factor that affects the stability of colloidal dispersions [10]. PS, PDI, and ZP values of PNP and SLN formulations are presented in Table **3**.

Particle sizes were found to be approximately 182 nm and 75 nm, respectively, in accordance with former studies [19, 20]. The Polydispersity Index (PDI) value defines size distribution, where a value closer to 0 defines homogenous size distribution and a value closer to 1 defines non-uniformity [21]. Formal guidelines classify dispersions with ZP values of ±0-10 mV as highly unstable, ±10-20 mV as relatively stable, ±20-30 mV as moderately stable, and >±30 mV as highly stable [22]. According to Table **3**, both PNP and SLN formulations can be considered as stable.

#### 4.3.2 Differential Scanning Calorimetry (DSC)

Differential Scanning Calorimetry (DSC) is a technique that quantifies the variation in heat flow rate between a substance and a reference material as a function of temperature, while both are subjected to a controlled thermal program [23]. DSC is one of the essential techniques for the characterization of polymeric and lipidic particles, as they offer valuable structural information about the particles dispersed [24]. DSC thermograms are presented in Fig. (**2**).

The complete disappearance of the IND peak in both PNP and SLN thermograms might be due to either homogenous matrix formation or dispersed IND within the amorphous polymer [22, 25].

#### 4.3.3 Fourier Transform Infrared (FT-IR) Spectrophotometry

FT-IR analysis is a method that relies on the selective absorption of light corresponding to the vibrational modes of specific chemical bonds. Consequently, FT-IR analysis provides concise information regarding the interactions that occur between the drug and the carrier during the nanoparticle formation stages by evaluating the changes in frequency and intensity of the spectral features compared to the FT-IR signals of the pure materials [22]. FT-IR chromatograms are given in Fig. (**3**).

When the FT-IR spectra of IND were examined, aromatic C-H stretching was observed in the 3200 cm^−1^ – 3000 cm^−1^ range, C-H stretching vibrations in the 3000 cm^−1^ – 2800 cm^−1^ range, C=O stretching vibrations in the 1800 cm^−1^ – 1600 cm^−1^ range, and symmetrical aromatic O-H stretching vibrations in the 1200 cm^−1^ – 1000 cm^−1^ range; all these peaks have been found to be consistent with the literature [26]. The changes in the peaks of IND in the physical mixture and the SLN formulation indicated the amorphous structure of IND. However, the appearance of specific peaks in all cases showed no chemical change between the carrier and the active substance, indicating IND to be encapsulated within the polymers and lipids [27].

#### 4.3.4 Nuclear Magnetic Resonance (NMR)

Nuclear Magnetic Resonance (NMR) spectroscopy is a widely recognized analytical method used to determine the structures of small and large molecules. The ^1^H (proton) NMR spectrum, which provides data on chemical shifts and coupling constants, also reveals insights into the quantitative relationships between intramolecular and intermolecular resonances [28]. ^1^H NMR spectra are given in Fig. (**4**).

Specific peaks of IND were observed in both the physical mixture and formulation spectra, though the intensity of these peaks varied depending on the amount of IND. An analysis of the spectra indicated no chemical changes, in accordance with the literature [19].

#### 4.3.5 Encapsulation Efficiency (%EE) and Loading Efficiency (%LE)

%EE value is of great importance in the characterization studies of nanoparticles. A high value of %EE ensures that an effective dose can be achieved with a lower amount [29]. %EE values of PNP and SLN are given in Table **3**.

In the literature, there are examples of encapsulation efficiency and loading efficiency values in between 20-40% using chitosan and cyclodextrins [30], approximately 3% in the case of solid lipid microparticles [31], and in between 30-40% with the use of different polymers and lipids [32]. All these varying results can change depending on the polymer used, the parameters (such as mixing speed, mixing temperature, and nanoparticle preparation methods), as well as the particle size. In our study, these values have been found to be approximately 31% and 22% for PNP and SLN, respectively, aligning with the literature.

#### 4.3.6 In Vitro Drug Release Test

The results of two different *in vitro* release studies are given in Figs. (**5** and **6**).

At pH 1.2, the amount released from PNP and SLN was observed to be significantly lower than that released from pure IND, leading to the conclusion that the release did not initiate at gastric pH. Following the transfer to pH 6.8, it was noted that the quantity released from PNP and SLN was less than that of the pure active substance. After 48 hours, IND exhibited a release of 99.29%, while PNP demonstrated a release of 34.92% in PBS medium and 48.54% in surfactant-containing médium, and SLN showed a cumulative release of 40.42% in PBS medium and 58.29% in surfactant-containing medium. Considering these results, it can be concluded that PNP and SLN formulations do not dissolve at gastric pH, regardless of the presence of surfactants, thereby potentially protecting against the adverse effects of IND, especially gastric mucosa irritation. Additionally, taking into account the prolonged release duration at intestinal pH, it can be suggested that the dosing frequency could be reduced.

According to the current literature, both polymers used in PNP formulation are able to postpone the release in the gastric media and prolong the release time by more than 24 h [15, 33]. In addition, Dynasan^®^116 has also been found to be able to prolong the release time [34, 35].

#### 4.3.7 MTT Assay

The effects of the studied groups on cell viability were evaluated based on time and concentration, and the results are presented in Fig. (**7**) and Table **4** along with statistical analyses.

According to international standards, a clear cytotoxic effect is indicated if the viability of cells exposed to the tested substance is less than 70% compared to the control cells not exposed to any substance [36]. In our study, cytotoxicity analyses conducted on HUVEC cells showed that IND started to exhibit cytotoxic properties at concentrations above 600 µM at the 24^th^ hour and above 200 µM at the 48^th^ hour. However, after being loaded into polymeric/lipidic nanoparticles, the cytotoxic effect of IND was observed to decrease. There are also studies in the literature indicating that the toxic effects of IND are reduced when loaded into nanoparticles [37–39]. In conclusion, all cytotoxicity analyses have indicated the IND-loaded polymeric/lipidic nanoparticles to effectively penetrate the cells and exhibit their activity in a concentration and time-dependent manner.

## CONCLUSION

The primary goal of this study was to extend the release duration of IND and reduce potential side effects by developing PNP and SLN formulations. Characterization studies have confirmed IND’s dissolution properties, thermal characteristics, FT-IR, and ^1^H NMR spectra, aligning with the literature data. %EE results have indicated adequate loading of IND. Optimum conditions for PNP and SLN formulations have been determined, and formulations have been prepared accordingly. Solid-state and particle characteristics of these formulations have also been analyzed, and the formulations have been found suitable for the study based on all characterization analyses. Considering the release results, it has been concluded that PNP and SLN do not dissolve at gastric pH, potentially protecting IND from side effects, while at intestinal pH, the extended-release duration could reduce dosing frequency. In cytotoxicity analyses, upon IND application for 24 and 48 hours, a linear correlation has been observed between drug concentration and cell viability, and the PNP formulation has been observed to exhibit fewer toxic effects among the formulations. This has proven the PNP nanocarrier to be safer for normal cells. In light of these results, further research plans include supporting the IND-loaded SLN and PNP formulations with *in vivo* studies and examining their stability.

## Figures and Tables

**Fig. (1):**
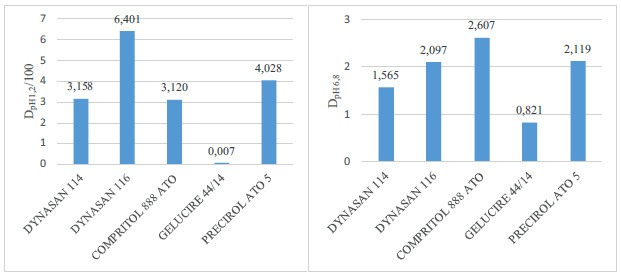
D values of the lipids at pH 1,2 and pH 6,8.

**Fig. (2):**
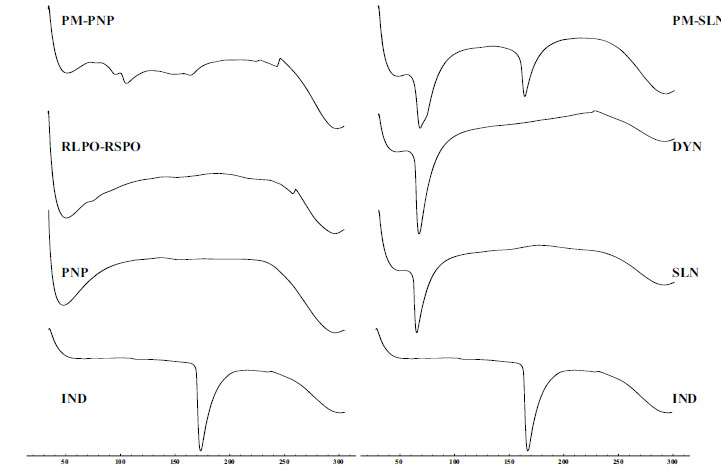
DSC thermograms of IND, physical mixture of PNP (PM-PNP), RLPO-RSPO mixture, PNP, physical mixture of SLN (PM-SLN), Dynasan^®^116 (DYN), and SLN.

**Fig. (3):**
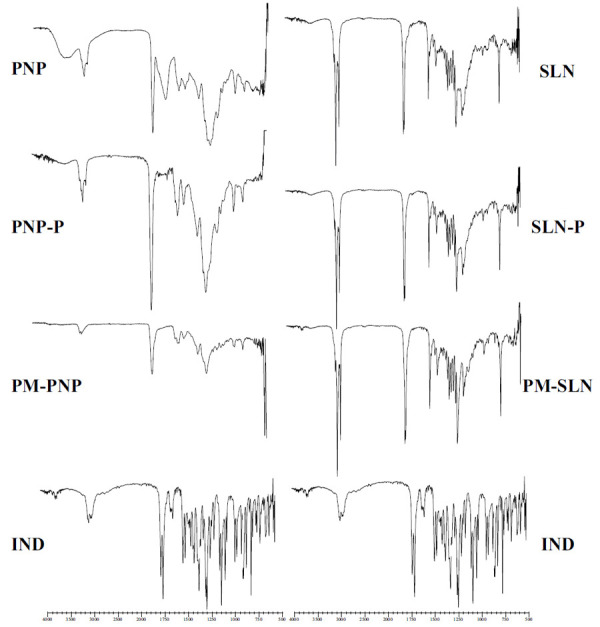
FT-IR chromatograms of IND, physical mixture of PNP (PM-PNP), placebo PNP formulation (PNP-P), PNP, physical mixture of SLN (PM-SLN), placebo SLN formulation (SLN-P), and SLN.

**Fig. (4):**
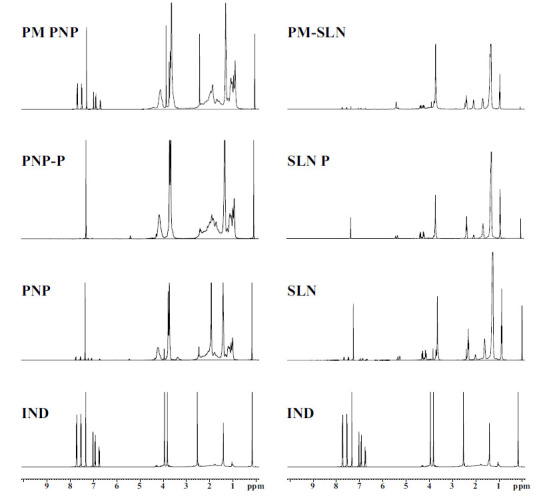
^1^H NMR spectra of IND, physical mixture of PNP (PM-PNP), placebo PNP formulation (PNP-P), PNP, physical mixture of SLN (PM-SLN), placebo SLN formulation (SLN-P), and SLN.

**Fig. (5):**
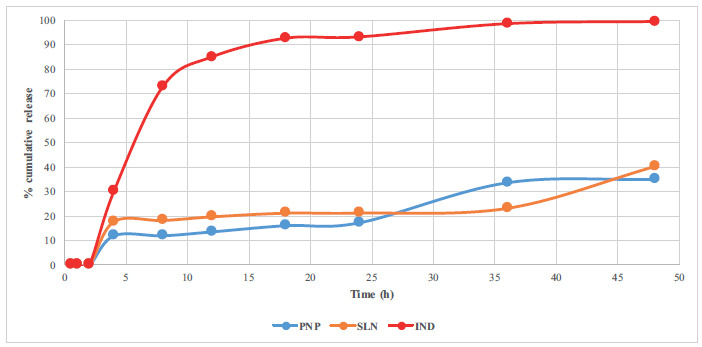
*In vitro* dissolution profile of pure IND, PNP, and SLN at pH 1,2 (first two hours) and in PBS without any surfactants.

**Fig. (6):**
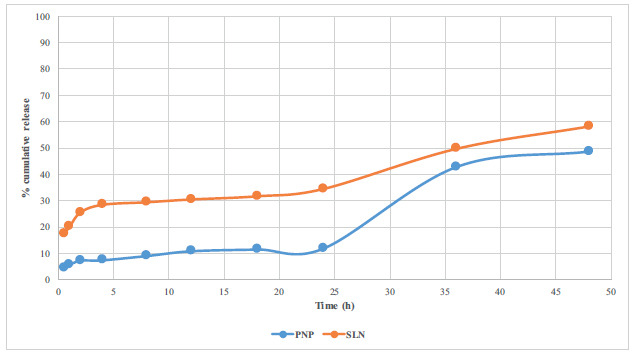
*In vitro* dissolution profile of PNP and SLN in PBS and Tween^®^80.

**Fig. (7):**
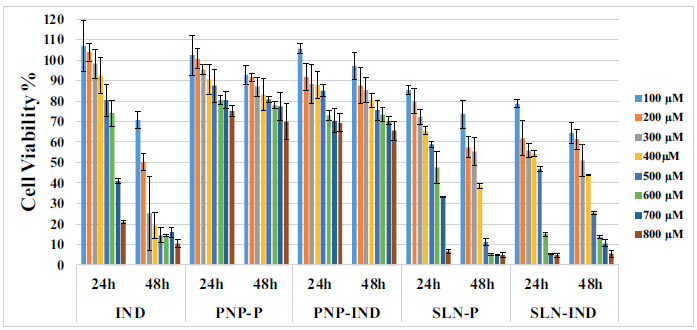
MTT assay results of pure IND, placebo PNP (PNP-P), PNP, placebo SLN (SLN-P), and SLN.

**Table 1:** The composition of PNP formulations.

**Formulation Code**	**IND**	**RLPO**	**RSPO**	**Aqueous Phase**
P-1	5 mg	50 mg	-	20mL bidistilled water: %1 Tween^®^80
P-2	5 mg	25 mg	25 mg	20mL bidistilled water: %1 Tween^®^80
P-3	5 mg	-	50 mg	20mL bidistilled water: %1 Tween^®^80

**Table 2:** The composition of the SLN formulations.

**Formulation Code**	**IND**	**Lipid**	**Span^®^80**	**Aqueous Phase**
TS-1	0.1 g	1 g	0.14 g	18.5 g bidistilled water: 0.26 g Tween^®^80
TS-2	0.1 g	1 g	0.12 g	18.6 g bidistilled water: 0.18 g Tween^®^80
TS-3	0.1 g	1 g	0.08 g	18.7 g bidistilled water: 0.12 g Tween^®^80

**Table 3:** PS, PDI, ZT, and %EE values of the formulations.

**NP**	**PS (nm)**	**PDI**	**ZP (mV)**	**%EE/%LE**
PNP	181.9 ± 17.4	0.452 ± 0.018	-18.8 ± 0.9	31.09 ± 0.04%
SLN	74.7 ± 1.0	0.368 ± 0.009	-18.4 ± 0.8	22.23 ± 0.17%

**Table 4:** IC_50_ values indicating the concentrations at which the studied groups exerted their effects on the cells (µM) (n=3).

**Time**	**IND**	**PNP-P**	**PNP**	**SLN-P**	**SLN**
24h	653	-*	-*	572	436
48h	201	-*	-*	318	305

Note: * IC_50_ values could not be calculated at the studied concentrations.

## Data Availability

The data that support the findings of this study will be available from the corresponding author upon reasonable request.

## LIST OF ABBREVIATIONS

%EE: Encapsulation Efficiency
%LE: Loading Efficiency
BCS: Biopharmaceutical Classification System
DSC: Differential Scanning Calorimetry
DYN: Dynasan^®^ 116
FT-IR: Fourier Transform Infrared Spectrophotometry
HPLC: High Pressure Liquid Chromatography
IND: Indomethacin
NMR: Nuclear Magnetic Resonance
NSAID: Non-steroidal Anti Inflammatory Drug
PDI: Polydispersity İndex
PM: Physical Mixture
PNP: Polymeric Nanoparticles
PS: Particle Size
RLPO: Eudragit^®^ RLPO
RSPO: Eudragit^®^ RSPO
SLN: Solid Lipid Nanoparticles
ZP: Zeta Potential
